# Locked plate versus modified clamp‐rod internal fixation for feline corpus ilium fractures: A comparative clinical study

**DOI:** 10.1111/vsu.70117

**Published:** 2026-05-14

**Authors:** Nahit Saylak, Sadık Yayla, Semih Altan, Berna Ersöz‐Kanay, Emine Çatalkaya

**Affiliations:** ^1^ Department of Surgery, Faculty of Veterinary Medicine Dicle University Diyarbakır Türkiye; ^2^ Department of Surgery, Faculty of Veterinary Medicine Dokuz Eylul University İzmir Türkiye

## Abstract

**Objective:**

To evaluate the applicability and clinical effectiveness of a modified clamp‐rod internal fixation (M‐CRIF) system compared with conventional locking plate osteosynthesis in the treatment of feline corpus ilium fractures.

**Study design:**

Prospective, controlled clinical study.

**Animals:**

Thirty‐six client‐owned cats with corpus ilium fractures.

**Methods:**

Cats were randomly assigned to two groups: Group I (M‐CRIF, *n* = 18) and Group II (locking plate, *n* = 18). All fractures were stabilized with a lateral approach. Radiographic healing, sacral index (SI), and complications were assessed at postoperative days 21, 45, 60, and 120. Long‐term outcomes were assessed using owner questionnaires addressing mobility and defecation.

**Results:**

Fracture union was achieved in all cats. Healing scores increased over time in both groups (*p* < .05). At day 45, Group I showed higher healing scores than Group II (*p* = .002). Sacral index narrowing was lower in Group I (*p* = .005). Implant‐related complications occurred in 22.2% (4/18) of cats in the plate group, including screw loosening and one revision surgery, whereas no screw loosening was observed in the M‐CRIF group. Preoperative neurological deficits were present in 22.2% of cats, decreasing to 5.5% postoperatively. Owner questionnaires indicated satisfactory mobility, although some discrepancies with clinical and radiographic findings were observed.

**Conclusion:**

The M‐CRIF system provided greater stability, fewer complications, and better preservation of the pelvic canal than locking plates.

**Clinical significance:**

This study is the first clinical evaluation of the M‐CRIF system in feline ilial fractures and demonstrates favorable outcomes, supporting its use as a reliable alternative to conventional plating.

**Abbreviations**
ALP: alkaline Phosphatase
ALT: alanine aminotransferase
AST: aspartate aminotransferase
BUN: blood urea nitrogen
CRI: continuous rate infusion
CRIF: clamp‐rod internal fixator
Hct: hematocrit
Hgb: hemoglobin
LCP: locking compression plates
M‐CRIF: modified clamp‐rod internal fixation
NSAIDs: nonsteroidal anti‐inflammatory drugs
PLT: platelet
RBC: red blood cell
SDs: standard deviations
SI: sacral index
SOP: string‐of‐pearls
SWP: screw‐wire‐polymethylmethacrylate
TPLO: tibial pleteau leveling osteotomy
VetFix: clamp‐rod internal fixator
WBC: white blood cell

## 1 INTRODUCTION

Pelvic fractures in cats are serious orthopedic injuries, frequently encountered following high‐energy trauma, such as high‐rise syndrome, traffic accidents, gunshot injuries, and bite wounds.^1^, ^2^ Pelvic fractures account for approximately 20% to 32% of long bone fractures in cats, and corpus ilium fractures constitute nearly 50% of these injuries.^3^, ^4^ Due to its role as a major weight‐bearing component of the pelvis, corpus ilium fractures affect both mechanical stability and pelvic alignment directly. Surgical management of os ilium fractures therefore typically involves open reduction and internal fixation using laterally placed plates. Other surgical techniques include dorsal or ventral ilial plating, compression of fracture fragments with Kirschner wires or lag screws, fixation with screw–wire–polymethylmethacrylate (SWP) composites, and external skeletal fixation systems.^5^, ^6^

However, literature reports indicate high implant‐related complication rates with these surgical techniques, particularly screw loosening, which has been documented in up to 62% of cases.^7^ This problem is especially common in cases where nonlocking implant systems are used.^3^ The cranial ilium has been identified as the region most prone to screw loosening, likely because of its low‐density cancellous bone structure, which compromises implant stability and increases the risk of failure.^8^, ^9^ To address these limitations, several modern implant systems, including tibial plateau leveling osteotomy (TPLO) plates, locking compression plates (LCP), String‐of‐Pearls (SOP) plates, and anatomical reconstruction plates have been developed in recent years. Nonetheless, sufficient screw placement remains a major constraint in narrow bone regions such as the ilium.^6^, ^10^, ^11^ Despite these advances, the search for an ideal implant system and surgical protocol for corpus ilium fractures continues.

The clamp‐rod internal fixator (CRIF), also known as a VetFix‐type fixator, originates from the AO Foundation framework. This versatile system has been used successfully to stabilize various fractures, including those of the femur, humerus, and vertebrae. It consists of a rod, clamps, and fixation screws.^12^

The present study aimed to evaluate the suitability of a newly designed modified locking CRIF system for feline corpus ilium fractures and to compare its stabilization capacity with that of conventional locking plates through clinical, radiographic, and sacral index (SI) assessments, together with long‐term outcomes based on caregiver‐reported scoring. For this purpose, the rod was contoured to match feline iliac anatomy, and the clamps were extended to allow placement of a greater number of screws. Unlike the conventional CRIF system, which lacks a locking mechanism, the modified system used in this study incorporated locking screw holes and heads within the clamps, which was hypothesized to enhance stabilization and reduce complications such as screw loosening.

To the best of our knowledge, this study represents the first clinical investigation evaluating the efficacy of a modified locking CRIF system for the treatment of feline corpus ilium fractures, providing clinically relevant data that may contribute to future advances in veterinary orthopedic practice.

## 2 MATERIALS AND METHODS

The study protocol was reviewed and approved by the animal experiments local ethics committee of the Dicle University Health Sciences Application and Research Center, Dicle, Türkiye (approval number: 08/08/2024‐01‐11), in accordance with national and institutional guidelines for the care and use of animals in research.

### 2.1 Materials

The study population consisted of 36 client‐owned cats of different breeds, sexes, body weights, and ages (1–5 years), which were presented to the Surgery Clinic of Dicle University Veterinary Faculty Animal Hospital with a history of trauma and radiographically diagnosed with corpus ilium fractures. In all cases, vital parameters including pulse, respiration, and body temperature were assessed initially. To determine surgical eligibility, hematological and biochemical evaluations were performed, including hemogram, glucose, blood urea nitrogen (BUN), aspartate aminotransferase (AST), alanine aminotransferase (ALT), and creatinine, in addition to radiographic examinations. Necessary medical treatments were administered, and preoperative radiographic measurements were obtained as required. All cats were also subjected to neurological evaluation both preoperatively and postoperatively, and any complications were recorded.

To ensure standardization, cats presenting with “tail‐pull” injuries, cauda equina or sacrococcygeal nerve dysfunction, loss of anal tone, absence of the perineal reflex, severe sciatic neurapraxia, loss of withdrawal reflex associated with sciatic nerve injury, and/or absence of deep pain perception in the paw, as well as those with multiple fractures accompanying corpus ilium fractures, were excluded from the study.

### 2.2 Preoperative procedure

Cat owners were informed about the study, and cases for which consent was obtained—including signed informed consent forms and, when preferred, group information disclosure—were included. Standard preoperative preparations, including shaving of the surgical area and aseptic disinfection, were performed. All cats were premedicated with medetomidine (80 μg/kg, IM) (Domitor, Orion Pharma, Finland) and ketamine (5 mg/kg, IM) (Ketasol, Richter Pharma, Austria). Following induction, endotracheal intubation was performed, and anesthesia was maintained with isoflurane (1.5% to 1.8%; 100 mL) (Adeka, Istanbul, Türkiye). For intraoperative analgesia, a continuous rate infusion (CRI) consisting of ketamine (12 mg), butorphanol (4.8 mg), and medetomidine (40 μg) diluted in 100 mL of 0.9% isotonic saline was administered intravenously at 5 mL/kg/h using an automated infusion pump, ensuring a standardized anesthetic protocol across all cases.

### 2.3 Operative procedure

The lateral surgical approach was performed for all cases in both groups. Each cat was positioned in lateral recumbency under general anesthesia to expose the corpus ossis ilii. The skin incision was initiated at the midpoint of the crista iliaca and extended caudally and distally to the level of the greater trochanter. The deep gluteal fascia was incised in line with the skin incision, allowing access through the intermuscular septum between the tensor fascia lata and gluteus medius muscles, while the cranial margin of the biceps femoris was also released. Retraction of the gluteus medius enabled visualization of the gluteus profundus and partial exposure of the corpus ossis ilii. Subperiosteal elevation of the gluteal musculature further exposed the crista iliaca, ala ossis ilii, and corpus ossis ilii, facilitating anatomical reduction of the fracture fragments.

**FIGURE 1:**
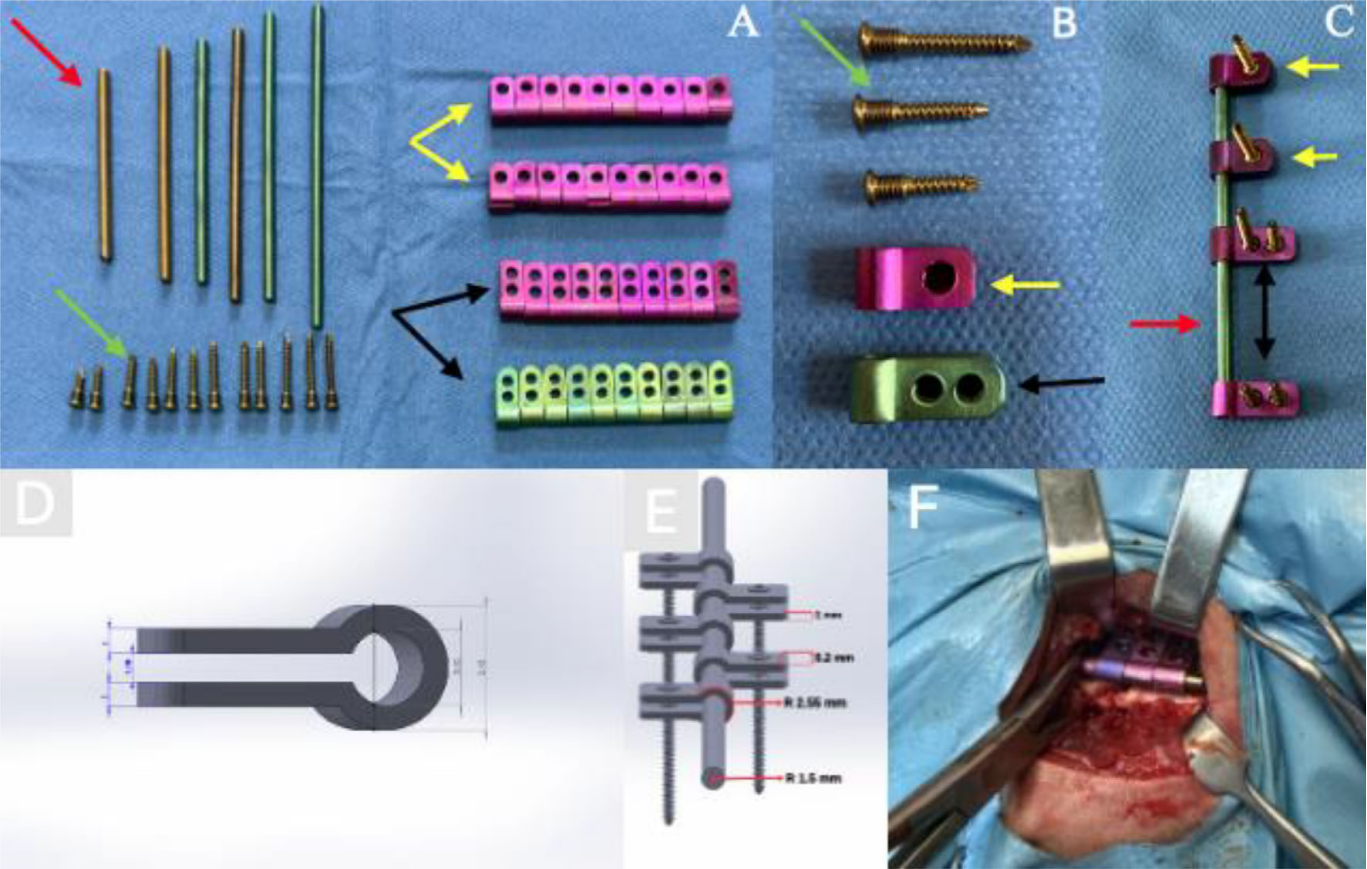
Implant set of the modified clamp‐rod internal fixation (M‐CRIF) system used in this study. (A) Orthopedic implant components of the M‐CRIF system. (B) Single and double clamps with specially designed screws long enough to connect and lock both clamp ends at the screw head. (C) Assembly of the implant system showing placement of the clamps on the rod and locking with screws (with the possibility of adjusting clamp orientation according to the surgeon's preference). (A–C) Rod (red arrows), single‐screw locking clamp (yellow arrows), double‐screw locking clamps (black arrows), and specially modified long screws designed to interconnect and lock both clamps at the screw head (green arrows). (D) Structure and measurements of the clamps used in this study. (E) Placement and dimensions of the clamps, rod, and screws in the M‐CRIF system. (F) Arrangement of the M‐CRIF system according to the anatomical shape of the ilium in an example case and demonstration of the system.

Following reduction, cats were randomly assigned to Group I (M‐CRIF, *n* = 18) or Group II (locking plate fixation, *n* = 18). In the M‐CRIF group, the rod component was contoured intraoperatively to match the anatomical curvature of the ilium. At least two extended clamps were applied cranial to the fracture line, each accommodating two locking screws, enabling placement of multiple screws to enhance fixation stability (Figure 1). In Group II, conventional 4–6 hole veterinary locking plates were applied to achieve stabilization.

Intraoperative monitoring (YSD 16 V‐vet veterinary monitor is Guangzhou, China) included continuous electrocardiography, pulse oximetry using a lingual probe, noninvasive blood pressure measurement, and temperature monitoring. Heart rate (100–160 bpm), systolic arterial pressure (110–160 mmHg), diastolic arterial pressure (55–100 mmHg), mean arterial pressure (100–110 mmHg), respiratory rate (16–40 breaths/min), SpO_2_ (95–100%), and body temperature (38–39°C) were recorded throughout the procedure to ensure adequate anesthetic and physiological stability.

### 2.4 Postoperative care and follow up

Following completion of the surgical procedures in both groups, cats were extubated after appropriate closure of the muscle and skin layers and transferred to the intensive care unit for recovery. Each cat was monitored until full awakening, and an appropriately sized Elizabethan collar was placed to prevent self‐trauma. Postoperatively, parenteral antibiotic therapy with cefazolin (20 mg/kg IM) (Deva, İstanbul, Türkiye) was administered for 7 days. Nonsteroidal anti‐inflammatory treatment (meloxicam, 0.05 mg/kg subcutaneous; Verano İlaç, Türkiye) was given for 3 days and extended when clinically required. To minimize potential gastrointestinal adverse effects of nonsteroidal anti‐inflammatory drugs (NSAIDs), famotidine (1 mg/kg orally; Sandoz İlaç, İstanbul, Türkiye) was administered once daily throughout treatment.

Radiographic evaluation was performed to assess bone healing, implant‐related complications (e.g., screw loosening, pelvic canal narrowing, and implant failure), and differences between M‐CRIF and locking plate fixation. Standardized radiographs were obtained preoperatively and at 21, 45, 60, and 120 days postoperatively. Healing was graded using a four‐point scale: 0 (no healing) = no callus or bridging, fracture line clearly visible; 1 (minimal healing) = small callus, fracture line still distinct; 2 (moderate healing) = substantial callus, fracture line partially obscured; and 3 (complete healing) = mature callus formation with radiographic union and absence of the fracture line.^13^, ^14^

Pelvic canal narrowing was further evaluated using SI measurements obtained from ventrodorsal radiographs immediately after surgery and at 6 weeks postoperatively.^10^ Measurements were repeated three times, and narrowing was categorized as mild (<10%), moderate (10% to 30%), or severe (>30%) according to previously defined criteria.^15^ Long‐term follow up (>6 months) was conducted via structured telephone interviews with cat owners. Mobility, including running and jumping ability, was assessed using a visual analog scale (1 = very poor activity, 5 = excellent activity).^14^ Questions addressing constipation frequency, severity of clinical signs, and veterinary interventions were included, and supplemental open‐ended questions were added at the clinician's discretion to clarify ongoing problems.

### 2.5 Statistical analysis

All data were analyzed using IBM SPSS Statistics v26.0 (IBM Corp., Armonk, New York). Continuous variables are presented as means ± standard deviations (SDs). The Shapiro–Wilk test was used to assess normality, and Levene's test was used to evaluate homogeneity of variances. Within‐group comparisons over time were performed using the Friedman test. When significant differences were identified, pairwise comparisons were conducted with the Wilcoxon signed‐rank test with the Bonferroni correction for multiple testing. Between‐group comparisons of non‐normally distributed data were analyzed using the Mann–Whitney *U*‐test. Categorical variables were assessed using the *χ* ^2^ or Fisher's exact test when more than 20% of cell counts were below five. A value of *p* < .05 was considered statistically significant.

## 3 RESULTS

Thirty‐six cats with corpus ilium fractures were randomly assigned into two groups: Group I (M‐CRIF, *n* = 18) and Group II (locking plate osteosynthesis, *n* = 18). In Group I, fixation was achieved using the modified clamp‐rod internal fixation (M‐CRIF) system, whereas cats in Group II underwent conventional locking plate stabilization.

Analysis of trauma etiology revealed that 28 cats (77.8%) sustained fractures due to falls from height, six (16.7%) were involved in traffic accidents, and two (5.5%) had fractures of unknown origin. Group I comprised eight males (44.4%; five neutered) and 10 females (55.6%; six spayed), and Group II included nine males (50.0%; four neutered) and nine females (50.0%; eight spayed).

The age distribution in Group I ranged from 13 months to 8 years (mean ± SD = 4.21 ± 2.18 years), whereas in Group II it ranged from 14 months to 7 years (mean ± SD = 3.94 ± 1.96 years). Body weight ranged from 3.14 to 5.32 kg in Group I (mean ± SD = 4.06 ± 0.61 kg) and from 3.01 to 5.01 kg in Group II (mean ± SD = 4.00 ± 0.56 kg).

The cats’ preoperative hematological and biochemical parameters are presented in Table 1 together with reference values.^16^ Most values in both groups remained within normal ranges. Mild deviations, including leukocytosis, borderline anemia, or slightly elevated ALT levels, were detected in five cats in the M‐CRIF group and three cats in the plate group. However, these alterations were not clinically significant and did not contraindicate surgery. All cats recovered uneventfully from anesthesia following the operative procedures.

**TABLE 1:** Hematological and biochemical parameters of cats with trauma (preoperative values)

Parameter	Reference range (cats)^a^	CRIF group (mean ± SD)	Plate group (mean ± SD)
RBC (×10^12^/L)	5.0–10.0	8.5 ± 1.2	8.3 ± 1.3
HGB (g/L)	80–150	122.0 ± 14.5	120.5 ± 15.2
HCT (L/L)	0.28–0.45	0.39 ± 0.05^a^	0.38 ± 0.04
WBC (×10^9^/L)	5.5–19.5	12.4 ± 3.1^a^	12.0 ± 3.4^a^
PLT (×10^9^/L)	300–800	540 ± 110	525 ± 120
Urea (mmol/L)	6.0–12.0	9.8 ± 2.1	10.1 ± 2.3
Creatinine (μmol/L)	71–212	135 ± 22	138 ± 25
ALT (U/L)	20–107	48 ± 12^a^	50 ± 13
AST (U/L)	15–66	32 ± 8	34 ± 9
ALP (U/L)	10–60	38 ± 9	36 ± 8
Total protein (g/L)	57–89	72 ± 6	73 ± 7

*Note*: Values are presented as mean ± standard deviation. Minor preoperative deviations were observed in five cats from the CRIF group and three cats from the plate group. Parameters marked with^a^ indicate mild abnormalities outside the reference ranges (e.g., mild leukocytosis, borderline anemia, or mild ALT elevation). These findings were not considered clinically significant.

Abbreviations: RBC, red blood cell; Hgb, hemoglobin; Hct, hematocrit; WBC, white blood cell; PLT, platelet; ALT, alanine aminotransferase; ALP, alkaline phosphatase; AST, aspartate aminotransferase.

^a^ Reference ranges were adapted from Fossum (2018).^16^

In both groups, bone healing scores improved over time (*p* < .05), with differences indicated by distinct superscript letters in the table. Between‐group comparison revealed a difference in bone healing scores on day 45 (*p* = .002). Sacral index narrowing (%) values also showed a difference between groups (*p* = .005). Long‐term caregiver questionnaire scores also differed between the groups (*p* < .05, Table 2).

**TABLE 2:** Mean ± standard deviation values of bone healing scores, SI (%) narrowing, and 6 month owner questionnaire results for Group I (CRIF) and Group II (plate fixation) with intergroup *p* values

Time	Group I (mean ± SD)	Group II (mean ± SD)	*p*
Day 21	1.00 ± 0.59^a^	0.89 ± 0.32^a^	.527
Day 45	2.72 ± 0.46b*	1.89 ± 0.83b*	.002*
Day 60	3.00 ± 0.00ᵇ	2.72 ± 0.67^c^	.080
Day 120	3.00 ± 0.00ᵇ	2.89 ± 0.32^c^	.163
SI (%)	1.85 ± 2.69	−7.19 ± 8.18*	.005*
Owner scale	0.89 ± 0.47	1.61 ± 0.85	.003*

*Note*: Different lower case letters (a, b, c) within the same row denote statistically significant differences within the same group over time (*p* < .05). An asterisk within the same column denotes a statistically significant difference between groups at the same time point (*p* < .05).

Abbreviations: SI, sacral index; SD, Standard deviations.

**FIGURE 2:**
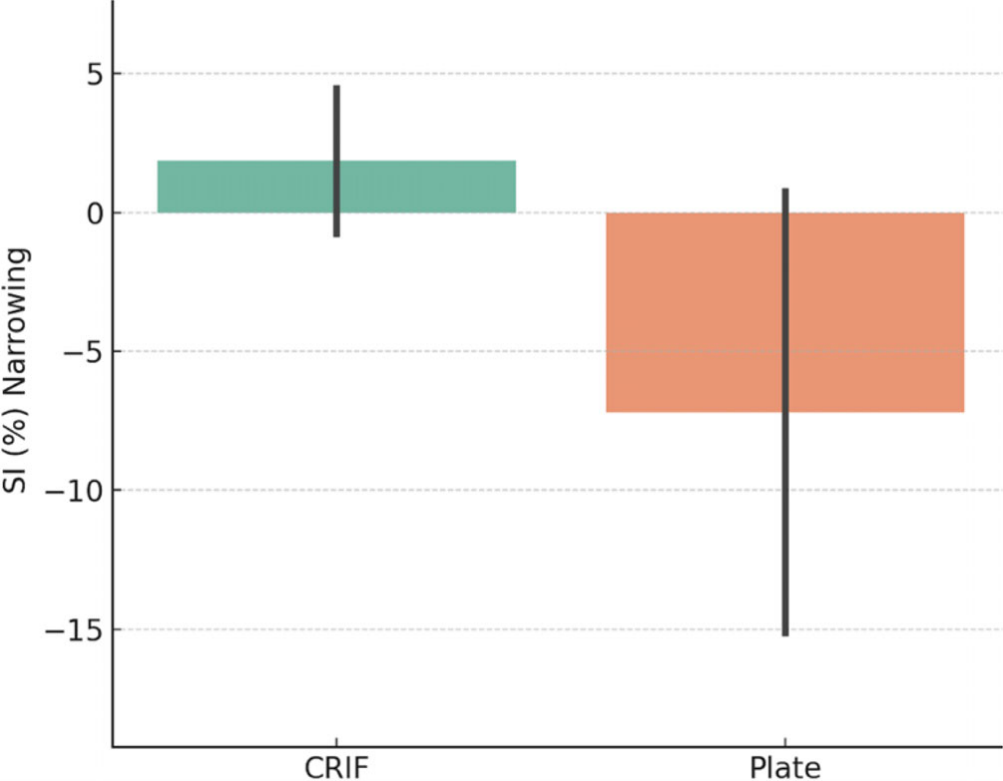
Comparison of sacral index (SI, %) narrowing values between the two groups. Comparative graphical representation of SI (%) narrowing values between the two groups. The modified clamp‐rod internal fixation (M‐CRIF) group demonstrated less canal narrowing (*p* = .005), with most cases classified as mild (< 10%) or showing slight widening, whereas the plate group exhibited higher rates of moderate narrowing (10% to 20%).

When SI values were specifically assessed, in Group I, two cats exhibited moderate narrowing (10% to 20%), 10 cats showed mild narrowing (<10%), and six cats demonstrated mild widening (<10%) of the pelvic canal. Similarly, in Group II, six cats had moderate narrowing (10–20%), seven cats showed mild narrowing (<10%), and five cats demonstrated mild widening (~10%) based on radiographic measurements (Figure 2).

Osteosynthesis performed with the M‐CRIF system (Group I) required a longer surgical time than conventional plate fixation (Group II), primarily because the implant system had to be adjusted intraoperatively to conform to the anatomical structure of the ilium. Although this duration decreased after several cases as the surgeon's familiarity with the technique improved, overall, plate fixation was completed in a shorter time. Despite the longer application period, anatomical reduction and stabilization of the fracture were found to be easier to achieve when the CRIF system was anatomically adapted to the bone.

**FIGURE 3:**
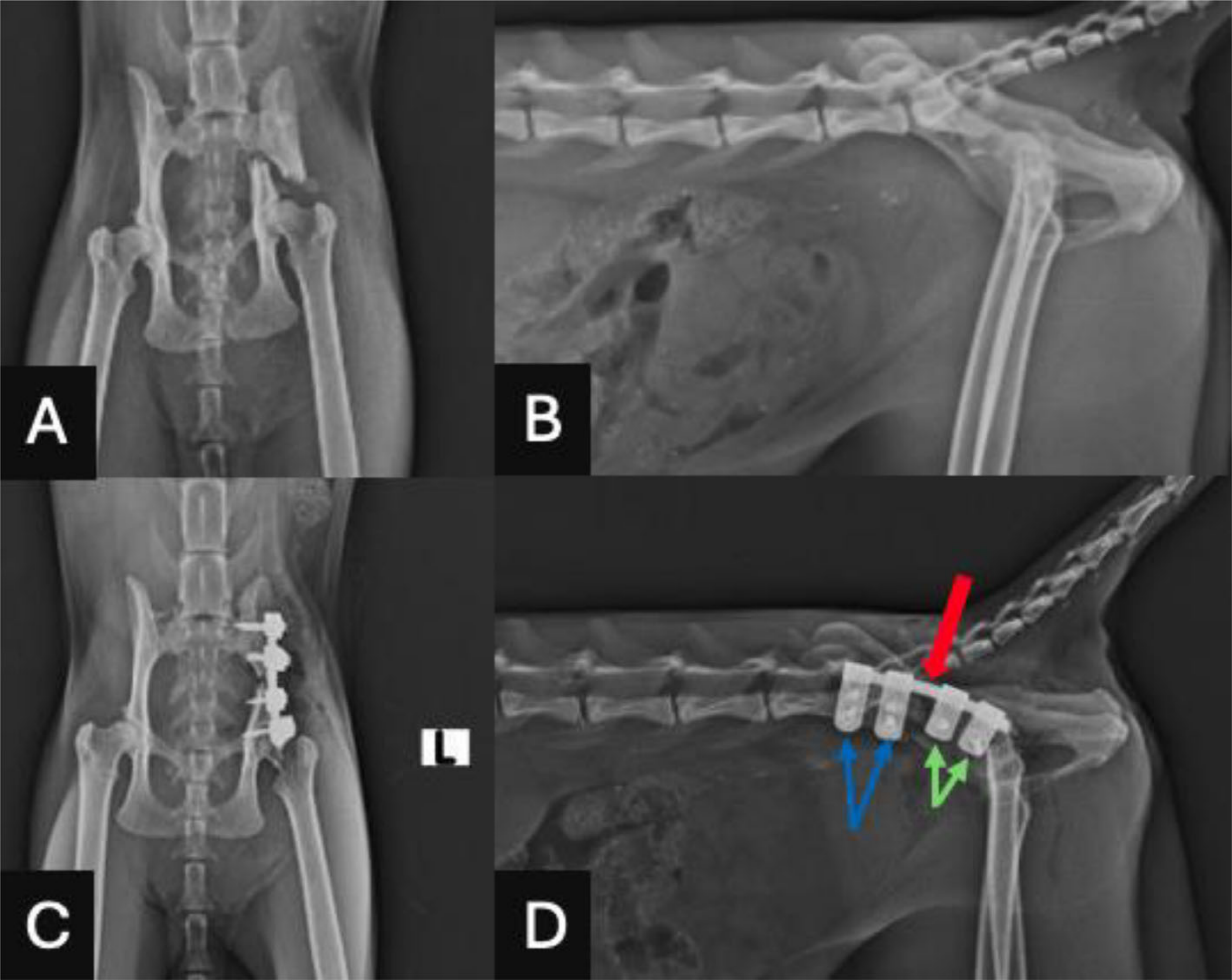
Radiographic projection of the modified clamp‐rod internal fixation (M‐CRIF) implant system in a case. (A) Preoperative V/D (Ventrodorsal) radiographic projection. (B) Preoperative L/L (Laterolateral) radiographic projection. (C) Postoperative V/D radiographic projection. (D) Postoperative L/L radiographic projection. The red arrow indicates the placement of the rod in the system. The blue arrows show the clamps that can be placed with two locking screws. The green arrows indicate the clamps that can be placed with a single screw.

The M‐CRIF system also allowed for the placement of four locking screws cranially and two locking screws caudally to the fracture line without difficulty (Figure 3). During application, after clamps were positioned on the caudal portion of the rod, the cranial placement enabled improved visualization, as only the clamps were in contact with the bone whereas other parts of the system remained elevated. This feature facilitated clear exposure of the fracture fragments and allowed more precise anatomical reduction.

**FIGURE 4:**
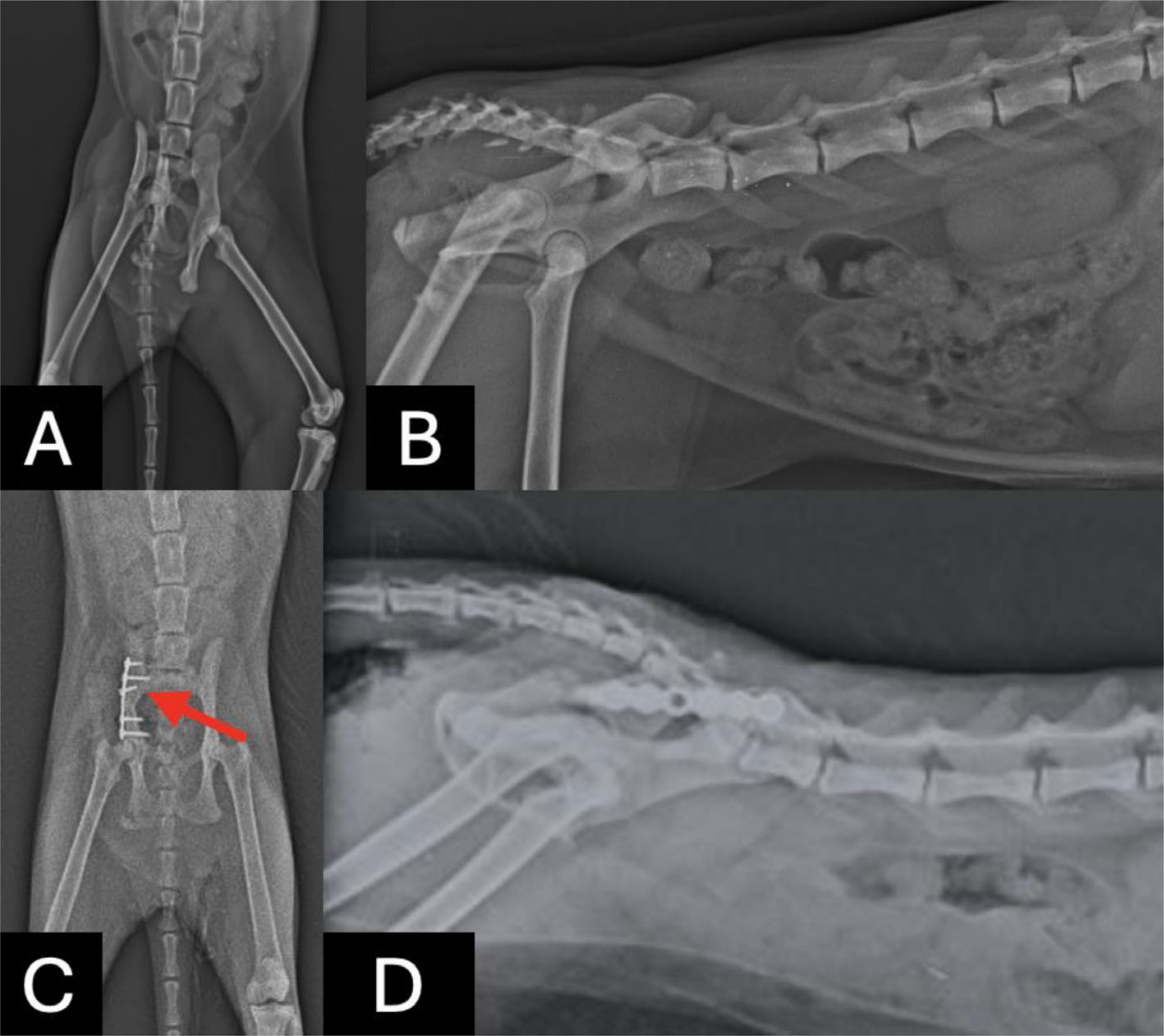
Radiographic projection of the plate implant system in an example case. (A) Preoperative V/D (Ventrodorsal) radiographic projection. (B) Preoperative L/L (Laterolateral) radiographic projection. (C) Postoperative V/D radiographic projection. The screw indicated by the red arrow should be directed differently and not locked to avoid being in contact with the fracture line. (D) Postoperative L/L radiographic projection.

The fundamental principle of corpus ilium fracture osteosynthesis in this study was that the clamps of the M‐CRIF system (Group I) could be positioned at the required region and angle of the bone, allowing screws to be inserted at a precise 90° angle and locked securely in place. In contrast, in the plate fixation group (Group II), the limited surface area of the fracture fragments generally permitted the application of no more than two screws per fragment. When more than four screws were required, angulated screw placement was often necessary to avoid penetrating the fracture line (Figure 4).

The relatively small size of the feline ilium, the restricted surgical field, and the cranial portion's spongious bone structure posed additional technical challenges during plate fixation. These anatomical constraints made both anatomical reduction and screw placement more demanding compared with the CRIF implant system, which offered greater flexibility and ease of application.

Fracture fragments ultimately achieved healing in all 36 cats included in the study. However, certain complications were observed. In Group I (M‐CRIF), one cat developed lameness due to the caudal portion of the rod being excessively long. At the third postoperative month, once fracture healing was confirmed radiographically, the implant was removed, after which the cat regained normal mobility without further complications, although mild wear of the proximal femur was noted. Aside from this case, no implant‐related problems were encountered, and importantly, no screw loosening was observed in any subject.

Neurological evaluation revealed that five cats in this group presented with deficits at the time of admission. Postoperatively, neurological problems persisted in only two cases, and at later follow up, only one cat continued to exhibit deficits. Clinical examinations confirmed that this residual lameness was not neurological in origin but was instead associated with the implant‐related complication mentioned above.

In the plate fixation group, implant‐related complications were observed in four cats, all of which developed screw loosening. Three of these cases achieved fracture healing without requiring revision surgery but one cat experienced loosening of all screws accompanied by displacement of the fracture fragments. In this case, revision surgery was performed. As the stability provided by a single laterally placed plate was deemed insufficient, an additional dorsal plate was applied, resulting in uneventful fracture healing, albeit with delayed consolidation.

Neurological deficits were present in three cats of this group at admission and persisted postoperatively in the same individuals. Two of these cases recovered within 2 weeks, whereas one cat continued to exhibit neurological deficits despite physiotherapy, with persistent weakening of the withdrawal reflex.

Three cats presented with mild constipation after the fourth postoperative month. Although medical treatment alleviated the problem in most cases, one cat continued to show recurrent signs at the eighth month, as reported in the owner‐based follow‐up survey.

In both groups, neither short‐term clinical evaluations nor owner‐based survey results revealed constipation‐related complications, except for one case in Group II. In this cat, the owner reported an increased frequency of attempts to defecate during the follow‐up period. A detailed clinical examination confirmed that this case belonged to the plate fixation group and coincided with an individual that developed screw loosening. Radiographic assessment demonstrated that this cat fell within the subgroup of six cases showing moderate SI narrowing.

## 4 DISCUSSION

To the best of our knowledge, this is the first clinical study to assess the application of a modified CRIF system in cats with corpus ilium fractures, where the design allowing two locking screws per clamp provided increased screw density and superior fixation stability compared with conventional plating.

In cats, various surgical techniques have been described for the stabilization of corpus ilium fractures, including open reduction with lateral, dorsal, or ventral plating; interfragmentary compression using Kirschner wires or lag screws; SWP composite; and/or external fixation systems.^5^, ^17^, ^18^ Among these, lateral plating is the preferred option, and the combined use of dorsal and lateral plates has also been suggested to enhance stability.^15^ In dogs, implant diversity is more advanced. In a recent study, Moi et al. designed a novel horseshoe‐shaped anatomic plate for iliac fractures and demonstrated through mechanical testing that this design improved screw purchase within the spongious structure of the ilium.^19^ Such findings highlight that innovative implant designs may enhance fixation strength. However, due to the smaller anatomical dimensions of the feline ilium, the application of such implants remains limited, and similar challenges are often addressed by combining lateral and dorsal plating. Likewise, in a recent retrospective study evaluating the use of SOP plates in feline pelvic fractures, canal narrowing and screw loosening were uncommon but consistently favorable outcomes could not always be achieved.^10^ These results emphasize the ongoing need for novel orthopedic implants, particularly for fractures involving the ilium. The present study represents the first clinical investigation describing a modified, versatile implant system that allows placement of a greater number of screws into the feline ilium. The results of this study suggest that increasing screw placement options may enhance implant stability and contribute to the development of implant designs specifically tailored to feline pelvic anatomy. Nevertheless, confirmation of these results and optimization of implant configurations for feline pelvises will require larger scale biomechanical and clinical studies.

In the present study, bone healing scores increased over time in both groups, indicating that each fixation method supported fracture healing. However, the statistically significant difference observed at day 45 can be attributed to implant‐related complications detected in the plate group. In this group, four cases developed screw loosening and one case required revision surgery due to complete screw failure and delayed union, which prolonged the healing process. In contrast, the M‐CRIF group experienced only a single implant‐related complication caused by an excessively long rod, which did not interfere with fracture healing. The absence of screw loosening or other major complications suggests that the modified system provided sufficient stabilization, contributing to more consistent healing outcomes. Similarly, Silveira et al. reported that implant‐related complications, particularly screw loosening, delayed fracture healing in distal femoral fractures.^20^ Overall, the results of this study suggest that both fixation methods are effective; however, the lower rate of implant‐related complications observed in the M‐CRIF group may offer a distinct advantage in early bone healing. The results also indicate that the CRIF technique may provide superior outcomes in midterm (day 45) fracture healing and in reducing postoperative pelvic narrowing compared to plate fixation, although bone healing scores in both groups were comparable in the later stages (≥3 months).

Radiographic evaluations revealed that all cats with iliac fractures exhibited a certain degree of pelvic canal narrowing, a finding consistent with previous studies reporting that medial displacement of the caudal iliac segment frequently reduces canal diameter.^15^ However, in the present study, no severe narrowing was observed in either group, and the degree of narrowing remained below the critical thresholds typically associated with recurrent constipation or predisposition to megacolon. Previous reports have suggested that severe pelvic canal narrowing may lead to recurrent constipation and related clinical signs.^15^ In fact, a canine study demonstrated that more than 30% of cases were classified as severe, with a mean collapse degree of 52% (range: 45% to 60%).^6^, ^15^, ^21^ In this study, the narrowing was particularly limited in the M‐CRIF group, and in some cases, even slight widening was observed, suggesting that this technique may offer a biomechanical advantage in preserving pelvic canal width. In contrast, narrowing was more pronounced in the plating group; although not clinically critical, the difference between groups was statistically significant (*p* < 0.05). These findings emphasize the importance of the surgical technique in maintaining long‐term pelvic canal function and indicate that the M‐CRIF system may represent a promising alternative for preserving functional canal width. Nevertheless, larger scale prospective clinical studies are warranted to clarify whether this advantage translates into reduced long‐term incidence of constipation and megacolon.

The use of locking implants for lateral osteosynthesis has been reported to result in lower rates of screw‐loosening complications. Specifically, in feline iliac fractures, screw‐loosening rates following lateral fixation with locking plates have been reported as low as 4%, compared with up to 50% with nonlocking plates.^5^ In the present study, no screw loosening was observed in the CRIF group, whereas four cases (22.2%; 4/18) in the plate group developed this complication. The spongious structure of the ilium allows for the placement of a greater number of screws in CRIF fixation, which, as previously suggested in the literature, may enhance screw stability and reduce the risk of loosening. Although it has been argued that eliminating motion at the plate–screw interface in locking plates creates a single‐beam construct that reduces the risk of screw loosening,^5^, ^22^ our findings appear more consistent with the view that pull‐out resistance is primarily determined by the “working length” of the screw.^23^ In the CRIF system, the flattened and irregular structure of the os ilium facilitates clamp positioning and enables screw insertion in optimal directions, allowing the use of longer working‐length screws. Despite comparable age and body weight distributions between groups, the use of longer screws in the CRIF group, combined with the absence of screw loosening in any case, suggests that working length may play a critical role in preventing this complication.

Neurological deficits were identified in 22.2% of cats during the preoperative period, a rate consistent with previously reported instances of approximately 23% in the literature.^15^ The anatomical course of the sciatic nerve, running medially to the iliac body and passing over the cranial ischium, places it at significant risk during trauma, explaining the relatively high incidence of concurrent neurological impairment. However, postoperative follow up revealed that only 5.5% of cases exhibited persistent neurological deficits, one of which resolved following implant removal, indicating a notably low rate of permanent complications. This favorable outcome may be attributed to the study's inclusion criteria, which limited cases to isolated corpus ilium fractures and excluded patients with severe neurological deficits. The reduction in the incidence of neurological deficits from 22.2% preoperatively to 5.5% postoperatively suggests that most injuries were likely neuropraxic in nature and that, with appropriate surgical stabilization and supportive therapy, the prognosis for recovery is generally favorable.

One of the limitations of the present study is that long‐term outcomes were based on owner questionnaires. However, Clarke and Bennett reported that assessments of lifestyle and mobility changes derived from owner‐completed questionnaires could yield results comparable to objective measurements. Similarly, Calvo et al. demonstrated that such questionnaires could serve as a practical alternative for evaluating functional outcomes following carpal and tarsal arthrodesis or surgical stabilization of sacroiliac luxations in cats.^24^, ^25^ In our study, owner questionnaires were generally consistent with clinical recovery and mobility outcomes; nonetheless, when clinical and radiographic findings were considered, owners tended to provide more optimistic evaluations. This observation is in line with the findings of Yap et al., who reported that despite identifying muscle atrophy in 7 out of 15 cats and pain on passive movement in four cats, all owners rated the procedure's outcome as satisfactory.^26^ Owner questionnaires may represent a valuable adjunct to clinical evaluation but they may also present discrepancies with clinical and radiographic data and should not be regarded as sufficient on their own. In this context, to minimize potential inconsistencies and ensure standardization, clinical and radiographic examinations were rigorously performed in all cases included in the present study.

## 5 CONCLUSION

This study demonstrates that the modified locking CRIF system provides significant clinical and radiographic advantages over conventional locking plate osteosynthesis in the treatment of feline corpus ilium fractures. The results highlight that the system enhanced implant stability, eliminated screw loosening complications, limited pelvic canal narrowing, and supported more consistent functional recovery. In particular, the challenges associated with screw stability in the spongious structure of the ilium were overcome by the clamp–rod configuration of the modified CRIF, which enabled the placement of a greater number and longer screws. To the best of our knowledge, this is the first clinical investigation in the literature to evaluate the modified locking CRIF system in feline corpus ilium fractures, suggesting that it may represent a reliable alternative by reducing complication rates. Nevertheless, further large‐scale, prospective, and long‐term studies are warranted to confirm its efficacy across different clinical scenarios.

## FUNDING INFORMATION

This study was supported by the Scientific and Technological Research Council of Türkiye (TÜBİTAK) under the 1002‐A Rapid Support Program (project no. 124O685). The authors gratefully acknowledge the support of the TÜBİTAK Project Office.

## CONFLICT OF INTEREST

The authors declare no conflicts of interest related to this report.
